# Binding Orientations and Lipid Interactions of Human Amylin at Zwitterionic and Anionic Lipid Bilayers

**DOI:** 10.1155/2016/1749196

**Published:** 2015-11-16

**Authors:** Zhenyu Qian, Yan Jia, Guanghong Wei

**Affiliations:** State Key Laboratory of Surface Physics, Key Laboratory for Computational Physical Sciences (Ministry of Education), and Department of Physics, Fudan University, Shanghai 200433, China

## Abstract

Increasing evidence suggests that the interaction of human islet amyloid polypeptide (hIAPP) with lipids may facilitate hIAPP aggregation and cause the death of pancreatic islet *β*-cells. However, the detailed hIAPP-membrane interactions and the influences of lipid compositions are unclear. In this study, as a first step to understand the mechanism of membrane-mediated hIAPP aggregation, we investigate the binding behaviors of hIAPP monomer at zwitterionic palmitoyloleoyl-phosphatidylcholine (POPC) bilayer by performing atomistic molecular dynamics simulations. The results are compared with those of hIAPP at anionic palmitoyloleoyl-phosphatidylglycerol (POPG) bilayers. We find that the adsorption of hIAPP to POPC bilayer is mainly initiated from the C-terminal region and the peptide adopts a helical structure with multiple binding orientations, while the adsorption to POPG bilayer is mostly initiated from the N-terminal region and hIAPP displays one preferential binding orientation, with its hydrophobic residues exposed to water. hIAPP monomer inserts into POPC lipid bilayers more readily than into POPG bilayers. Peptide-lipid interaction analyses show that the different binding features of hIAPP at POPC and POPG bilayers are attributed to different magnitudes of electrostatic and hydrogen-bonding interactions with lipids. This study provides mechanistic insights into the different interaction behaviors of hIAPP with zwitterionic and anionic lipid bilayers.

## 1. Introduction

Many human diseases, such as type II diabetes mellitus, Alzheimer's disease, Parkinson's disease, and Huntingdon's disease, are associated with protein aggregation and amyloid formation [1–4]. In type II diabetes mellitus, the cytotoxicity is most likely related to membrane damage, which leads to attrition of insulin-producing *β*-cells [5–7]. The primary component of islet amyloid and actual fibril-forming molecule is human islet amyloid polypeptide (hIAPP or amylin), a 37-residue peptide which is synthesized in pancreatic islet *β*-cells and cosecreted with insulin. The normal physiological role of hIAPP is still unclear, but it is believed to have correlations with gastric emptying, suppression of food intake, and glucose homeostasis [8–10].

Like other amyloidogenic peptides, it is believed that hIAPP forms amyloid deposits via a nucleation-dependent aggregation pathway characterized by a lag phase associated with the formation of a nucleus [11]. The early intermediates were reported to play important nucleating roles in hIAPP fibrillation and NMR experimental studies have shown that these intermediates are large in size [12–16]. Increasing evidence suggests that the prefibrillar intermediates, such as oligomers and protofibrils, are the primary toxic species to trigger pathological processes [12–14], while mature amyloid fibrils themselves exert only a minimal cytotoxic effect on pancreatic *β*-cells [17–19]. When hIAPP interacts with membranes, its aggregation can be dramatically accelerated. The intermediate oligomers as well as the fibrillization process can disrupt membrane integrity and thereby cause toxicity [20–22].

Experimental studies reported that monomeric hIAPP exhibits predominantly a random coil conformation in aqueous solution, and residues 8~19 of the peptide transiently adopt an *α*-helical structure [23–25]. In the presence of lipid membranes, hIAPP initially binds to the membrane in a helical state [21, 26]. Electron paramagnetic resonance (EPR) spectroscopy study showed that the *α*-helical region of hIAPP at neutral pH spans residues 9~22 and is oriented parallel to the surface of large unilamellar vesicles containing negatively charged lipids [27]. An earlier nuclear magnetic resonance (NMR) study demonstrated that residues 7~17 and 21~28 adopt helical structure in sodium dodecyl sulfate (SDS) detergent micelles [28]. As the concentration of membrane-bound peptides rises, hIAPPs cooperatively convert from *α*-helical intermediates to *β*-sheet aggregates [24, 29–31]. It was reported that the N-terminal 1~19 fragment of hIAPP is primarily responsible for membrane interaction, while the amyloidogenic 20~29 fragment is mainly responsible for fibrillar aggregates [26, 32, 33]. These hIAPP aggregates may reconstitute membranes and form amyloid ion channels, which would mediate ion transport and destabilize the cell ionic homeostasis [5, 34–36]. Nonselective ion channel activity of polymorphic hIAPP double channels was reported recently by experimental and MD simulation studies [37, 38]. In addition, previous experiments demonstrated that the toxic hIAPP and its variants primarily interact with the curved regions of the membrane [39], and lipids of phosphatidylethanolamine (PE) type exhibit intrinsic curvature strain [40], indicating that membrane curvature may play very important roles in the polymerization of hIAPP. Pore-like structures and channel activities are also reported in the studies of cytotoxicity induced by A*β* and PrP [35, 41]. Lipid composition was suggested to be one of the major factors that influence hIAPP aggregation, and the presence of membranes that contain negatively charged lipids, such as phosphatidylglycerol (PG) or phosphatidylserine (PS), can significantly accelerate the aggregation process [13, 27, 29, 42, 43]. However, the effect of lipid composition on the structures and orientations of hIAPP on membrane surface at atomic level are not well understood.

On the computational side, several studies have investigated the structures of monomeric/oligomeric species of full-length and the fragments of hIAPP in aqueous solution [44–51] and in membrane environment [52–55]. Our previous study demonstrated that monomeric hIAPP has a preferential orientation on anionic palmitoyloleoyl-phosphatidylglycerol (POPG) bilayers [56]. As a first step to understand how lipid composition modulates the aggregation of full-length hIAPP, in this study, we investigate the binding orientation and membrane interaction of hIAPP at zwitterionic palmitoyloleoyl-phosphatidylcholine (POPC) bilayer by conducting multiple atomistic MD simulations and then compare the results with those obtained at anionic POPG bilayers. Through the comparison of binding behaviors and lipid interactions of monomeric hIAPP at POPC and POPG bilayers, we try to understand at atomic level the membrane-modulated hIAPP aggregation in the membrane environment.

## 2. Materials and Methods

### 2.1. Peptide-Membrane System

The amino acid sequence of hIAPP is KCNTATCATQ^10^RLANFLVHSS^20^NNFGA ILSST^30^NVGSNTY, with the Cys2 and Cys7 forming a disulfide bond that constrains the first four residues in a disordered hairpin loop. To mimic experimental conditions, the N-terminus was charged and the C-terminus was amidated. At neural pH, the side chains of Lys1 and Arg11 are positively charged. We constructed the zwitterionic membrane using POPC lipids because phosphatidylcholine (PC) is the most abundant phospholipids in pancreatic islets [57]. This model membrane consists of 2 × 64 POPC lipids and the initial atomic coordinates were obtained from a previous computational study of a neat POPC lipid bilayer by Tieleman and Bentz [58]. Na^+^ and Cl^−^ ions were added to neutralize the system and provide an additional 0.1 M salt concentration.

Numerous experimental studies reported that hIAPP adopt predominantly *α*-helical structure when initially bound to the membrane [21, 23–27]. Consistently, a recent spectroscopic study reported that hIAPP transiently sample an *α*-helical structure in solution that becomes fully stabilized when bound to the surface of a membrane containing negatively charged lipids [59]. As the time scale for protein folding at water/membrane interface is on the order of milliseconds to seconds, it is still out of reach to sample conformational transition from a random coil to a helical structure at physiological temperature. Therefore, we took one of the NMR-derived conformations (pdb ID: 2KB8) solved in SDS micelles [60] as the starting point of our MD simulations, as done in previous studies [54, 61–63]. This NMR-derived conformation is not a fully folded *α*-helix. It consists of a helix running from residue 5 to 28 and disordered structures for the N-terminal residues 1–4 and the C-terminal residues 29–37 [60]. The choice of an *α*-helical conformation as a starting structure in our study is an MD strategy to speed up the simulation outcome as the time for coil-to-helix transition at water/membrane interface is on the order of milliseconds to seconds. Although the interfacial folding of shorter peptides could be probed by replica-exchange molecular dynamic (REMD) simulations as we did for hIAPP(1–19) in our recent study [64], it would be too expensive for a peptide of 37 residue with current computational resources.

**Figure 1:**
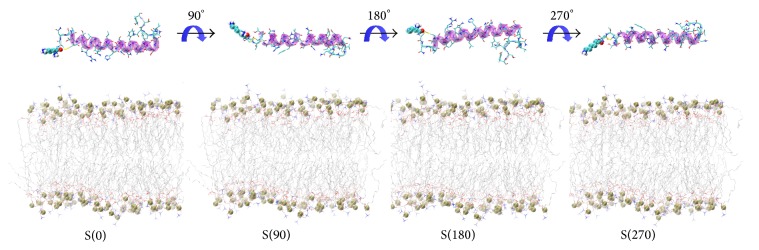
Four different initial states of simulated hIAPP-POPC systems. The side chain of residue K1 in S(0) points toward the POPC membrane surface. The other orientations of hIAPP are generated by rotating the hIAPP helix in (a) by 90°, 180°, and 270° around the helix axis. The other three different initial states are labeled as S(90), S(180), and S(270), according to the rotation angle. The peptide is shown in cartoon representation, with the helix (residues 5–28) in purple, the coil in orange and the other secondary structure in cyan. Bond representation is given for each amino acid residue, except for K1 in van der Waals (vdW) representation. The lipids are shown in grey line representation and phosphorus atoms as tan spheres. For clarity, counterions and water molecules are not shown.

The helical region of hIAPP monomer was initially orientated parallel to the membrane surface with a minimum distance ≥1.4 nm between the peptide and the POPC bilayer. We chose four different starting orientations of hIAPP with respect to POPC bilayer surface (see Figure 1) so that the peptide was allowed to adjust itself before adsorption to the bilayer surface. In the initial state of S(0), the side chain of residue K1 points toward the membrane surface. The initial state S(90), S(180), and S(270) were generated by rotating the hIAPP peptide in S(0) by 90°, 180°, and 270° around the axis of the helix, respectively. Each hIAPP-membrane system was immersed in a SPC water [65] box.

### 2.2. Simulation Details

All MD simulations have been performed in the isothermal-isobaric (*NPT*) ensemble using the GROMACS 3.3.3 software package [66]. Currently, several force fields are available for protein-lipid system, such as GROMOS87/Berger, OPLS-AA/Berger, AMBER99sb-ILDN/SLIPIDS, GROMOS54A7, and CHARMM36 [67–73]. The GROMOS force field and Berger force field have been widely used for proteins and lipids, respectively. Berger force field borrowed the standard parameters of the GROMOS force field for bonds, valence angles, improper dihedrals, and the dihedral angles in the headgroup region of lipids. Thus, the combination of GROMOS force field with Berger force field is appropriate for peptide-membrane system. The lipid is described with the Berger force field [68], and the peptide is described with GROMOS87 force field [67]. The POPC parameters used in this study have the correction on the double bond suggested by Bachar et al. [74]. The time step used in MD simulations is 2 fs. Peptide bonds are constrained by the LINCS algorithm [75] and water geometries are constrained by SETTLE [76]. Berendsen's coupling protocols were used for pressure and temperature couplings [77]. The pressure is maintained at 1 bar using a semi-isotropic scheme in which the lateral and perpendicular pressures are coupled separately with a coupling constant of 1.0 ps and a compressibility of 4.5 × 10^−5^ bar^−1^. The temperature of the system is maintained at 310 K with a coupling constant of 0.1 ps, above the gel-liquid crystal phase transition temperature ~270 K of POPC and POPG lipid bilayers [78, 79]. Long-range electrostatic interaction is calculated using the Particle Mesh Ewald (PME) method [80] with a real space cutoff of 1.2 nm, as recommended for membrane simulations, especially for those involving charged lipids [81]. van der Waals interaction is calculated using a cutoff of 1.4 nm. Three independent 120 ns MD runs were carried out for each system starting from the four initial states, using different initial velocity distributions.

### 2.3. Analysis

We analyze the MD trajectories using our in-house-developed codes and the GROMACS facilities. The *z*-position of each amino acid residue is described by the *z*-component of the main chain or side chain centroid with respect to the average *z*-position of the phosphorus atoms. A residue is considered to be the one closest to the bilayer surface if the *z*-position of its centroid is the smallest among all the residues. The number of hydrogen bonds (H-bonds) is calculated using Gromacs tool g_hbond. A H-bond is considered to be formed if the distance between N (H) and O is less than 0.35 (0.25) nm and the angle of N–H…O is greater than 150°. This geometrical criterion for hydrogen-bond formation is widely used in many previous studies [55, 63, 82–87]. The interaction energy *U* between peptide and lipid is computed using the GROMACS tools g_ener and mdrun-rerun (using the formula *U*_inter_ = *U*(peptide + lipid) − *U*(peptide) − *U*(lipid)). To examine the effect of hIAPP on the ordering of bilayer surface, we calculate the thickness of lipid bilayer and the order parameter *S*_CD_ of the lipid acyl chain (sn-1). The thickness of lipid bilayer is estimated by the average of the phosphorus-to-phosphorus distance [82]. All of the snapshots are displayed using the VMD program [88]. Trajectory data of hIAPP monomer at POPG bilayer membrane are obtained from our previous study [56]. The initial relative orientations of the peptide with respect to the membrane surface are the same for POPC and POPG lipid bilayers.

## 3. Results and Discussion

### 3.1. Adsorption of hIAPP Monomer from Aqueous Solution to the POPC Bilayer Is Mostly Initiated from the C-Terminal Residues

**Figure 2:**
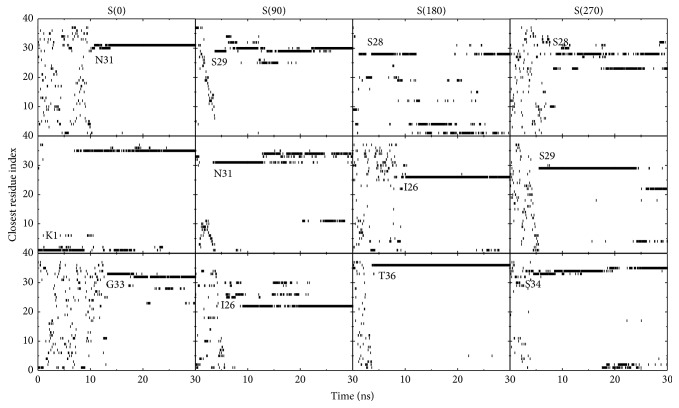
Index of residues closest to the POPC membrane surface as a function of time. A residue is considered to be the one closest to the bilayer surface if the *z*-position of its centroid is the smallest relative to the average *z*-position of the phosphorus atoms. In water solution, the closest residue index varies with time; once the peptide adsorbs to the membrane surface, the index of the residue closest to the POPC bilayer rarely changes.

We have calculated the *z*-position of the centroid of each residue with respect to the POPC bilayer surface and present the closest residue index as a function of time in Figure 2. We observe that in 11 out of 12 MD runs, the C-terminal residues are observed to adsorb to the POPC bilayer surface prior to the N-terminal residues, namely, the adsorption of hIAPP monomer is initiated from the C-terminal residues. This membrane adsorption behavior of hIAPP may be attributed to the dipole-dipole interaction between the polar residues (such as Ser34, Asn35, and Thr36) and the zwitterionic POPC lipids. In our previous study [56], we observed that the positively charged residues K1 and R11 in the N-terminal region have a preference to adsorb to the anionic POPG lipid bilayer. Previous experimental studies reported that the N-terminal residues are involved in the membrane entry of hIAPP peptide [32], and result in membrane damage at high peptide concentration [19, 89]. Our results show that the adsorption process of hIAPP monomer to POPC and POPG bilayer membranes is distinct, which may lead to different binding behaviors and may influence the aggregation of membrane-bound hIAPPs.

**Figure 3:**
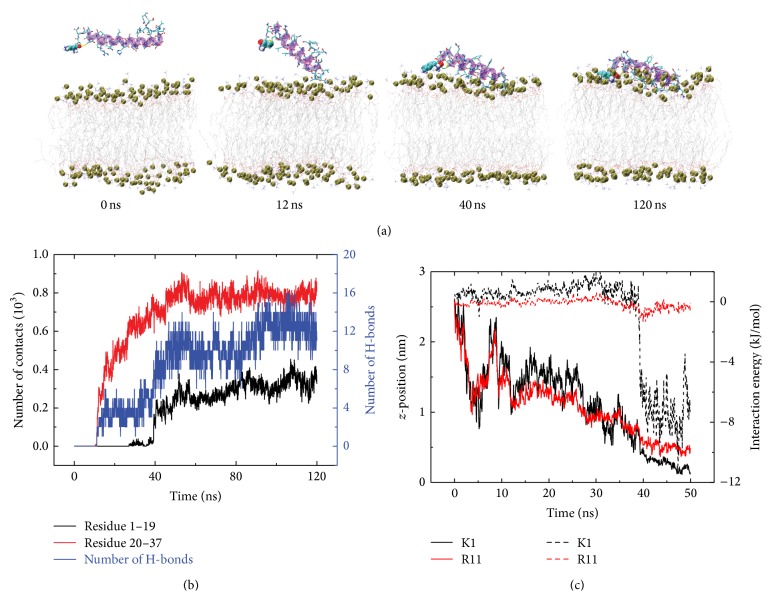
Detailed analysis of a representative MD trajectory of hIAPP adsorption to POPC bilayer surface, starting from the initial state S(0). (a) Snapshots at *t* = 0, 12, 40, and 120 ns. Each snapshot is displayed using the same representations as those used in Figure 1. (b) Time evolution of the number of contacts and the number of H-bonds between hIAPP peptide and the POPC lipid bilayer. (c) Time evolution of *z*-position and interaction energy between lipid bilayer and the negatively charged residues K1 (black) and R11 (red). The solid and dashed lines correspond to *z*-position and interaction energy, respectively. We only present the data of first 50 ns in order to show the initial adsorption process clearly.

To give the detailed adsorption process, we show in Figure 3 the snapshots at different time points and the time evolution of the contact number and hydrogen bond number between residue 1~19/20~37 and POPC headgroups in a representative MD run started from the initial state S(0). It can be seen from Figure 3 that in the initial state, the hIAPP monomer is placed in water parallel to the POPC bilayer with the side chain of residue K1 pointing toward membrane surface. The contact numbers between the C-terminal residues 20~37 and POPC lipids increase with simulation time. At *t* = 12 ns, the C-terminal residues 20~37 adsorb to the membrane surface prior to the N-terminal residues. Then, it takes tens of nanoseconds for residues 20~37 to adjust their side chains. At *t* = 50 ns, hIAPP monomer is mostly adsorbed to membrane surface and stays on the bilayer surface in the remaining 70 ns of MD simulation. The larger contact number of C-terminal residues 20~37 with POPC lipids with respect to the N-terminal residues 1~19 indicates that the C-terminal residues 20~37 interact with the membrane more strongly than the N-terminal residues 1~19. As seen from Figure 3(b), the adsorption process is accompanied by the formation of H-bonds between hIAPP and the headgroups of POPC lipids.  Figure 3(c) gives the time evolution of the *z*-position of the positively charged residues (K1 and R11) and their interaction energy with POPC bilayer within the first 50 ns of MD simulation. It is observed that K1 and R11 approach to the membrane surface at ~50 ns (solid line in Figure 3(c)), while the C-terminal residues reach to the bilayer surface within 15 ns (see Figure 2). In addition, the interaction energy between the POPC bilayer and residue K1/R11 is positive during the first 40/30 ns of simulation, reflecting the existence of repulsive interaction between the positively charged residues and the POPC lipids in the beginning of the simulations. It is known that a POPC lipid molecule is composed of a positively charged choline, a negatively charged phosphate group and hydrophobic fatty acids. Although the POPC lipid has no net charge, the positively charged choline is located closer to the membrane-water interface than the negatively charged phosphate group (see Section 3.3 for more details about the location of choline and phosphate groups), which leads to net repulsive interactions during the adsorption process. This net repulsive interaction disfavors the N-terminal residues to adsorb first to the membrane surface, which explains the observed C-terminal-initiated adsorption behavior (see Figure 2). These results provide the first step of hIAPP-membrane interactions. Interestingly, both insertion and some helical folding were observed in our recent REMD study on hIAPP(1–19) peptide [64]. Based on the results of our REMD study [64], we deduce that the next step of hIAPP-bilayer interaction might proceeds through insertion of partially ordered structures followed by helical folding within the interface [90, 91]. However, the exact mechanism remains clearly to be determined.

### 3.2. hIAPP Monomer Displays Multiple Binding Orientations at Zwitterionic POPC Bilayers, Different from Its Binding Behavior at Anionic POPG Bilayers, Which Has Only One Preferential Binding Orientation

**Figure 4:**
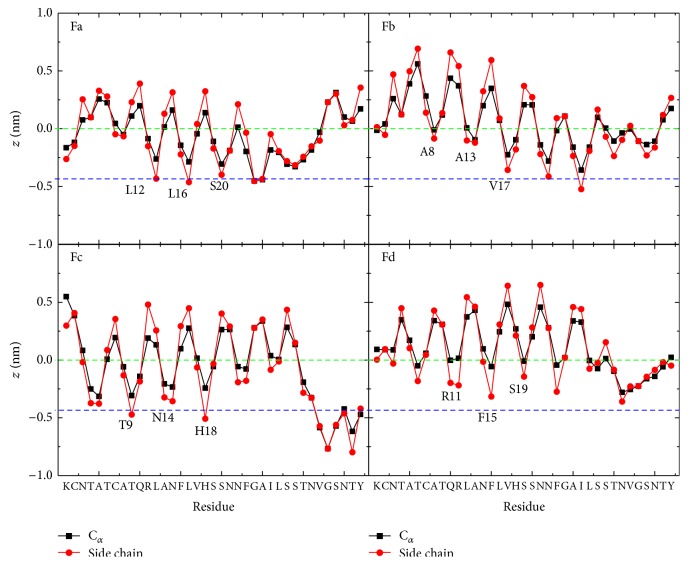
The *z*-positions of the C_*α*_ atom (black) and the side chain centroid (red) of each amino acid residue of hIAPP at POPC bilayer surface. For each membrane binding orientation (Fa, Fb, Fc, and Fd), the *z*-position is averaged using the last 20 ns data of each MD run (see Table 1). The green and blue dashed lines correspond, respectively, to the average position of phosphorus atoms and that of carbon atoms of the ester group of lipids, between which is the headgroup region of the upper leaflet. The residues that have a smaller *z*-position relative to their adjacent residues in the helical 8–20 region are labeled in the figure.

To investigate the peptide orientation at the membrane-water interface, we plot in Figure 4 the *z*-positions of C_*α*_-atom and side chain centroid of each residue. As experimental results have shown that the membrane-bound hIAPP monomer adopts an *α*-helix spanning residues 8~19 [23–25], we classify the membrane binding orientation of hIAPP into four different of orientations (labeled as Fa, Fb, Fc, and Fd) according to *z*-positions of the residues in the core helix region (residues 8~19). Our recent MD study showed that the side chains of residues R11, F15, and S19 insert more deeply into the anionic POPG bilayer than their neighboring residues [56]. This binding resembles the binding orientation Fd of hIAPP at the zwitterionic POPC bilayer (Figure 4). However, four different membrane binding orientations are observed for hIAPP at zwitterionic POPC bilayers, with almost equal probability. We give in Table 1 the initial states and the final hIAPP orientations in each MD run. As seen from Table 1, hIAPP with the same initial orientation can lead to different final binding orientations, and those with different initial orientations can lead to the same final binding orientation. These results suggest that hIAPP monomer adopts multiple binding orientations at POPC membrane surface independent of its initial orientation.

**Figure 5:**
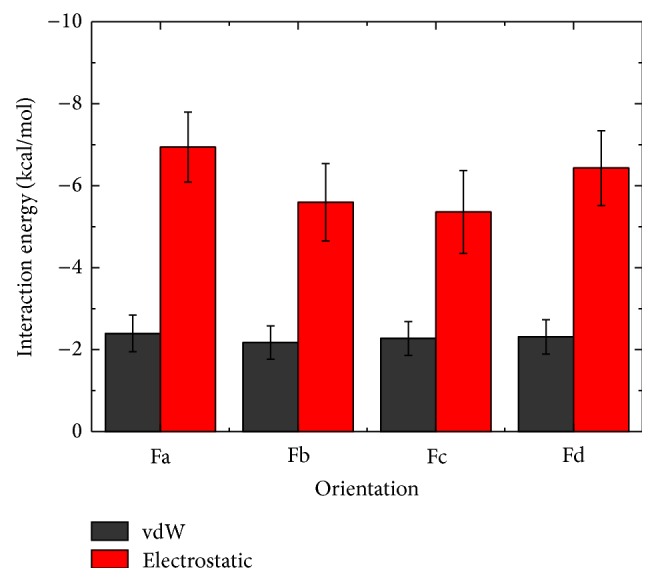
hIAPP-POPC interaction energy (per lipid) for hIAPP with four different binding orientations. The interaction energy is decomposed into the vdW (black) and electrostatic (red) component, calculated using the final 20 ns data of each MD run.

To identify the important interactions that stabilize each binding orientation of hIAPP at POPC bilayer, we plot in Figure 5 the interaction energy between a peptide and a lipid bilayer (per lipid). The interaction energy is decomposed into electrostatic and van der Waals (vdW) components. As seen from Figure 5 that hIAPPs with four binding orientations have nearly the same vdW interaction energy with lipids, and the electrostatic interaction energy is also similar. However, the electrostatic interaction is much stronger than vdW interaction, indicating that the former plays a dominant role in stabilizing the binding of hIAPP monomer to the POPC lipid bilayer although the net charge of a POPC lipid is zero. Different from our result, a recent MD study by Zhao et al. reported that the electrostatic and vdW interaction energy between hIAPP ion-channel and a DOPC bilayer are quite similar [37].

It is of particular interest to note that for the four different binding orientations, the peptide-lipid electrostatic interaction energy overlaps with each other (see the error bar of the average value). The small differences in total peptide-lipid interaction energy (the same vdW + similar electrostatic interaction energy) allow multiple binding orientations of hIAPP at the POPC lipid bilayer; that is to say, hIAPP has no preferred binding orientation to the membrane surface.

**Figure 6:**
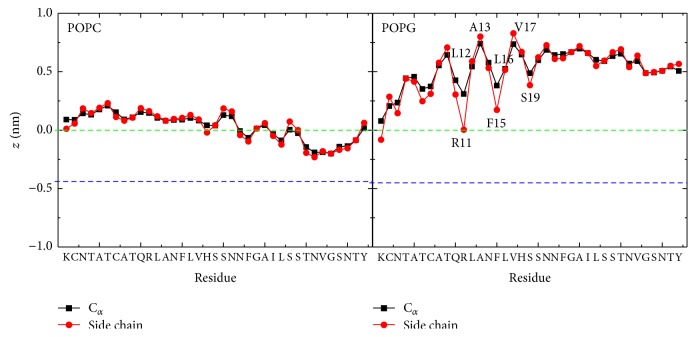
Comparison of averaged *z*-positions of the C_*α*_ atom (black) and the side chain centroid (red) of each amino acid residue at the POPC (left) and POPG (right) membrane surface. The green and blue dashed lines correspond, respectively, to the average *z*-position of phosphorus atoms (*z* = 0) and carbon atoms of the lipid ester groups.

In order to compare the binding orientations of hIAPP at POPC bilayer with those at POPG bilayers, we plot in Figure 6 the *z*-position of each amino acid residue of hIAPP relative to the average *z*-position (*z* = 0) of phosphorus atoms, averaged over the last 20 ns of twelve independent 120 ns MD runs. It is seen that, at the POPC membrane surface, the *z*-position of each residues is very close to the position of lipid phosphorus atoms (green dotted line). The similar *z*-position of all residues reflects the uncertainty of peptide orientations. At the POPG bilayer surface, the positively charged residues K1 and R11 anchor to the bilayer surface by electrostatic interactions, and the *z*-position gradually increases from the N-terminal to C-terminal residues. The *z*-position of residues in Figure 6 shows that the *α*-helix region (residues 5~19) of hIAPP is parallel to the POPG bilayer surface, with residues R11, F15, and S19 pointing to the lipid bilayer while the hydrophobic residues L12, A13, L16, and V17 exposed to the water, revealing a preferred binding orientation at anionic POPG bilayers.

### 3.3. hIAPP Monomer Exhibits Distinct Lipid Interactions at Zwitterionic POPC and Anionic POPG Bilayer Membranes

Previous experimental studies reported that the N-terminal residues of hIAPP are mainly responsible for membrane insertion and the C-terminal residues for fibrillar aggregation [26, 32]. Figure 6 shows that the whole hIAPP peptide binds tightly to the lipid headgroups of POPC, with the C-terminal residues buried below the phosphorus atoms, thus restraining the flexibility of the C-terminal residues. When hIAPP monomer binds to the POPG membrane, the C-terminal residues 20~37 are immersed in the water solution. This allows the amyloidogenic 20~29 region to have more freedom than the residues inside lipid bilayer, which facilitate peptide-peptide interaction. On the other hand, the helix-helix association of hIAPP is believed to proceed before the *β*-sheet formation of the disordered C-terminal region [29, 92]. Our MD simulations show that hIAPP monomer exposes the hydrophobic face of the amphipathic helical region to the solvent when binding to POPG lipid bilayer surface. These exposed hydrophobic residues may promote hIAPP aggregation. The preferential binding orientation of hIAPP may be attributed to the strong electrostatic interaction between the N-terminal positively charged residues K1 and R11 and the negatively charged POPG lipid headgroups. When hIAPP monomers interact with POPC lipid bilayers, their multiple binding orientations would reduce the solvent exposure probability of hydrophobic residues, thus disfavoring the peptide-peptide association. These results are consistent with experimental reports that negatively charged membranes can promote hIAPP aggregation [29, 43].

**Figure 7:**
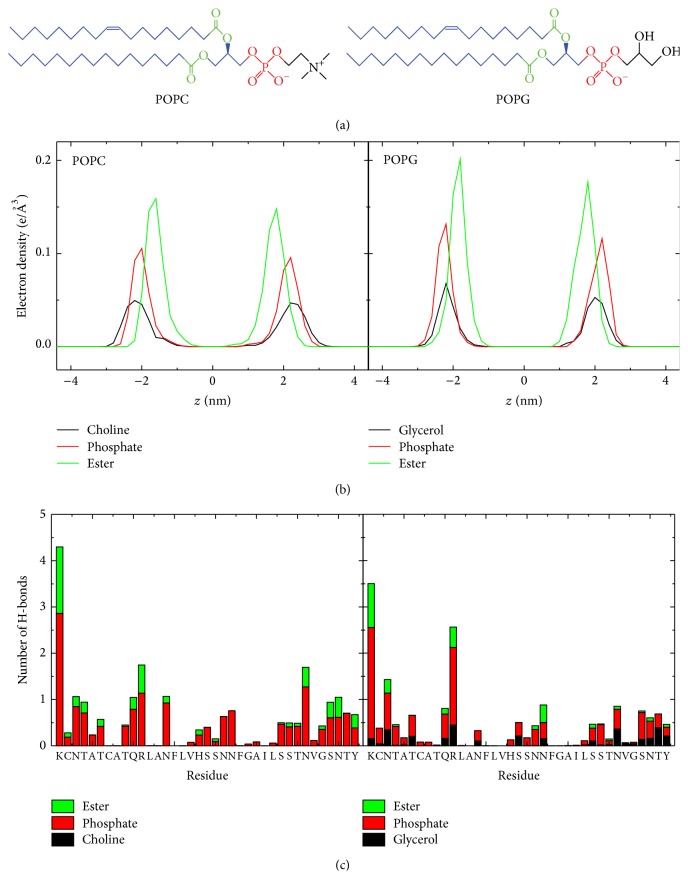
(a) Molecular structures of a POPC/POPG lipid molecule. Different lipid groups are colored differently: choline/glycerol in black, phosphate group in red, ester group in green, and other carbon atoms in blue. (b) Electron density profiles of the lipid choline/glycerol, phosphate, and ester groups. Here, we set the *z*-position of bilayer at zero (*z* = 0). (c) Number of H-bonds formed between each amino residue of hIAPP and the three different groups in lipid heads: choline (POPC)/glycerol (POPG), phosphate, and ester groups.

Through detailed structural analysis, we find that although the chemical components of POPC and POPG lipids are similar, the locations of these components in the membrane are different. We plot in Figure 7 the electron density of POPC and POPG along the membrane normal (i.e., *z*-axis), as done in a previous study of lipid bilayers [82]. It shows that the POPC ester, phosphate, and choline groups are located in turn from the membrane center (*z* = 0) to the water solution, while the POPG phosphate and glycerol groups are nearly at the same depth in the membrane with ester groups buried deeper. In addition, the peak value of each POPC headgroup component is smaller than that of POPG, and the average area per lipid of POPC membrane is higher than that of POPG (61.6 ± 0.7 Å^2^ versus 54.6 ± 0.6 Å^2^), consistent with previous computational and experimental studies [82, 93, 94]. Overall, the headgroup region of the POPC bilayer is less compact than that of the POPG bilayer, which is helpful for the insertion of hIAPP monomer into POPC membrane. The symmetric distributions of POPC/POPG lipid atoms in the upper and lower leaflets of the bilayer reveal that hIAPP monomer does not cause membrane disruption, in agreement with experimental observations [29, 95].

We also calculate the number of hydrogen bonds formed between hIAPP monomer and different groups of the POPC and POPG membrane.  Figure 7(c) shows that hIAPP monomer forms hydrogen bonds most with the phosphate groups and less with the ester groups and least with the glycerol groups. The formation of hydrogen bonds between the POPC ester groups and residues N31, S34, N35, and Y37 of hIAPP (see Figure 7(c)) allow hIAPP to interact with the hydrophobic lipid tails, which may result in the C-terminal residues insertion deep into POPC bilayer. It is noted that the POPC choline groups can not form hydrogen bonds with hIAPP monomer, while the POPG glycerol groups can. The formation of H-bonds between hIAPP and the glycerol groups, together with the formation of H-bonds between hIAPP and the phosphate groups, would stabilize the specific binding orientation of the peptide at POPG bilayer. On the other hand, the formation of these H-bonds would constrain hIAPP monomer at the POPG membrane surface and thus hinders the peptide inserting into the bilayer, which might be helpful for the peptide-peptide association through the water-exposed hydrophobic residues. This result is resembling the results reported for an antimicrobial peptide MSI-78 by NMR and fluorescence experiments where the peptide insertion was measured with the variation of the PC : PG ratio, showing that the peptide inserts more deeply in zwitterionic lipid bilayers than that in anionic lipid bilayers [96].

### 3.4. hIAPP Monomer Alters the Local Thickness but Displays Negligible Perturbation on the Integrity of POPC Membranes

**Figure 8:**
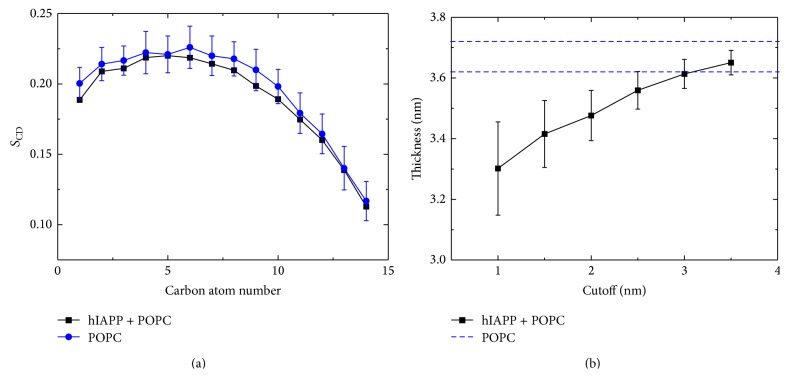
Influence of membrane-bound hIAPP monomer on the tail ordering of POPC lipids. (a) Lipid tail order parameter *S*_CD_ of acyl chain 1 (sn-1). In the calculation, the lipids within 1 nm (minimum distance) from any nonhydrogen atom of hIAPP peptide are considered. We also give the *S*_CD_ of a neat POPC lipid bilayer for comparison, obtained from the last 10 ns of a 100 ns MD run. (b) Local membrane thickness for lipids within six different cutoffs from hIAPP peptide. The thickness is calculated using the average *z*-position of the phosphorus atoms in the upper leaflet and that in the lower leaflet. The upper and lower bounds of the thickness of a neat POPC bilayer membrane (36.7 ± 0.5 Å) are plotted in blue dashed lines, consistent with that in [69].

The toxicity of hIAPP and membrane disruption are suggested to be associated with hIAPP-membrane interactions [29, 95]. To examine the effect of membrane-bound hIAPP monomer on the POPC membrane, we calculate the lipid tail order parameter *S*_CD_ of acyl chain 1 (sn-1) and the local membrane thickness (see Figure 8). The *S*_CD_ value is calculated by the formula *S*_CD_ = 0.5〈3cos^2^⁡*θ* − 1〉, where *θ* represents the angle of the C–H bond vector (in the simulation) or the C–D bond vector (in the experiment) with the bilayer normal. The angular brackets indicate averaging over lipids and over time [97]. As seen from Figure 8, the averaged *S*_CD_ value is within the error bar of the neat POPC lipid bilayer, implying that lipid interaction of hIAPP monomer does not disturb the membrane integrity. The calculated local thickness of lipid bilayers in Figure 8(b) using different cutoff demonstrates that hIAPP-lipid interaction influences the local thickness of POPC bilayer. The influence can be neglected when the cutoff is larger than 3 nm. These results suggest that the binding of hIAPP monomer at the POPC membrane surface has negligible disturbance on the integrity of the POPC bilayer, which provides atomic-level evidence that membrane-bound hIAPP monomer does not cause membrane disruption [29, 95]. However, it is expected that when the concentration of membrane-bound peptide reaches a critical value, the hIAPP-lipid interaction may cause membrane disruption.

## 4. Conclusions

In this study, we have investigated the binding orientation and lipid interaction of hIAPP monomer at the zwitterionic POPC bilayer by carrying out multiple MD simulations. We have also examined the influence of lipid composition on lipid binding by comparing results of hIAPP at anionic POPG bilayer. We have found that hIAPP monomer adopts multiple orientations at POPC bilayers while it has a preferential orientation at POPG bilayers. The specific binding orientation of hIAPP at POPG bilayer allows the hydrophobic residues exposed to water, thus facilitating peptide-peptide association by hydrophobic interactions. Our results also show that the hIAPP monomer forms more hydrogen bonds with the headgroups of POPG lipids, which constrains hIAPP monomer to the POPG bilayer surfaces, while the formation of less H-bonds allows hIAPP (especially the amyloidogenic C-terminal residues) inserts deep into POPC bilayer, thus reducing the probability of peptide-peptide interaction via solvent-exposed hydrophobic residues. Our studies provide atomic-level information of the binding behavior of hIAPP and the effect of lipid composition on hIAPP-membrane interactions, which may improve our understanding of membrane-mediated hIAPP aggregation.

## Figures and Tables

**Table 1:** Membrane binding orientations of hIAPP monomer at POPC bilayer. Our MD runs start from four different initial states S(0), S(90), S(180), and S(270). For each initial state, there are three independent 120 ns MD runs, and the final binding orientations are identified using the data of last 20 ns. According to the residues binding to the bilayer surface, four different binding orientations are identified and they are named as Fa, Fb, Fc, and Fd. These four binding orientations are given in Figure 4.

	Run1	Run2	Run3
S(0)	Fb	Fd	Fd
S(90)	Fa	Fd	Fb
S(180)	Fa	Fb	Fc
S(270)	Fd	Fc	Fb
